# *Arabidopsis* myrosinases link the glucosinolate-myrosinase system and the cuticle

**DOI:** 10.1038/srep38990

**Published:** 2016-12-15

**Authors:** Ishita Ahuja, Ric C. H. de Vos, Jens Rohloff, Geert M. Stoopen, Kari K. Halle, Samina Jam Nazeer Ahmad, Linh Hoang, Robert D. Hall, Atle M. Bones

**Affiliations:** 1 Department of Biology, Norwegian University of Science and Technology (NTNU), Realfagbygget, NO-7491 Trondheim, Norway; 2 Plant Research International, Wageningen UR, Droevendaalsesteeg 1, 6708 PB Wageningen, The Netherlands; 3 Netherlands Metabolomics Centre, Einsteinweg 55, 2333 CC Leiden, The Netherlands; 4 RIKILT, Wageningen UR, Akkermaalsbos 2, 6708 WB Wageningen, The Netherlands; 5 Department of Mathematical Sciences, NTNU, Trondheim, Norway; 6 Department of Botany, University of Agriculture Faisalabad (UAF), Pakistan; 7 Cellular and Molecular Imaging Core Facility (CMIC), Laboratory for Electron Microscopy, NTNU, Trondheim, Norway; 8 Laboratory of Plant Physiology, Wageningen University, P.O. Box 16, 6700 AA Wageningen, The Netherlands

## Abstract

Both physical barriers and reactive phytochemicals represent two important components of a plant’s defence system against environmental stress. However, these two defence systems have generally been studied independently. Here, we have taken an exclusive opportunity to investigate the connection between a chemical-based plant defence system, represented by the glucosinolate-myrosinase system, and a physical barrier, represented by the cuticle, using *Arabidopsis* myrosinase (thioglucosidase; TGG) mutants. The *tgg1*, single and *tgg1 tgg2* double mutants showed morphological changes compared to wild-type plants visible as changes in pavement cells, stomatal cells and the ultrastructure of the cuticle. Extensive metabolite analyses of leaves from *tgg* mutants and wild-type *Arabidopsis* plants showed altered levels of cuticular fatty acids, fatty acid phytyl esters, glucosinolates, and indole compounds in *tgg* single and double mutants as compared to wild-type plants. These results point to a close and novel association between chemical defence systems and physical defence barriers.

Plants exist in a dynamic environment where they face multiple biotic and abiotic stresses. Their survival depends on how well they can adapt and defend themselves against these challenges^1^,^2^. To cope with environmental stress, plants have adopted systems to protect cellular activities and maintain whole plant integrity. Several plant defence systems, such as the ‘plant cuticular defence’ and the ‘glucosinolate-myrosinase defence’ are well known^3^,^4^,^5^,^6^,^7^,^8^. However, both of these defence systems have been described in terms of their independent modes of action, with just a few exceptions. This is in terms of their interactive effect relating to insect herbivores by extracting glucosinolates from the leaf surface^9^,^10^,^11^, or glucosinolates possibly originating from deeper leaf layers, herbivores penetrating the wax layer and perceiving compounds like isothiocyanates in deeper layers or through stomata^12^. Glucosinolates are structurally diverse phytochemicals produced throughout the Brassicaceae, including *Arabidopsis thaliana* and *Brassica* crop species^8^,^13^,^14^. They comprise one component of the dual glucosinolate-myrosinase system, in which myrosinase thioglucosidase (TGG) (EC 3.2.3.147) catalyses glucosinolate breakdown into various biologically active molecules upon tissue disruption or insect attack^14^,^15^,^16^.

The cuticle acts as a barrier for water and solutes and regulates gas exchange when stomata are closed. Its ecological importance is clear in preventing plant desiccation under water deficit conditions and by providing the first physical barrier to herbivorous insects and pathogens^17^,^18^,^19^,^20^,^21^. The cuticle is considered to play an important role in plant drought tolerance by delaying the onset of cellular dehydration stress under water deficit conditions^7^,^22^.

Under changing climatic conditions, discovering novel links between interactions of plants with local climatic and environmental stress factors is currently a major and challenging research goal. Since both the glucosinolate-myrosinase system and the plant cuticle are well known for playing a role in plant abiotic/biotic defence, we have initiated research to find potential links between these two defence systems. In the aboveground plant parts of *Arabidopsis*, two functional myrosinase-encoding genes, *TGG1* and *TGG2*, have been reported^23^,^24^. *TGG1* is expressed in guard cells and phloem cells^15^,^16^,^25^ and and the TGG1 protein is highly abundant in guard cells^4^. In contrast, *TGG2* is only expressed in phloem-associated cells^16^.

The glucosinolate-myrosinase system, mostly known as a defence agent against insects and pathogens, has been shown to be important for key abscisic acid (ABA) responses of guard cells^4^. The *tgg1* mutant showed a hyposensitive response to ABA inhibition of guard cell localized inward K^+^ channels and delayed stomatal opening. Additionally, methyl jasmonate, which induces stomatal closure^5^,^26^,^27^, down-regulated *GUS* expression in transgenic *Arabidopsis* plants carrying a β-glucuronidase (GUS) fused to 2.5 kb TGG1 promoter (pBITGG1-GUS)^15^,^28^. These findings were the stimulus for us to seek insights into the potentially deeper role of the glucosinolate-myrosinase system in plant cuticular defence. Moreover, the cuticle being a barrier between the plant and environment provides physical defence, and the well-established role of glucosinolate-myrosinase system against insect herbivores and pathogens a chemical defence, raises enormous interest to find link between these two kind of defence systems. This is due to their concerted effect on insect herbivores through extraction of glucosinolates from the leaf surface, or glucosinolates probably originating from deeper leaf layers, herbivores penetrating the wax layer, and perceiving compounds like isothiocyanates in deeper layers or through stomata^10^,^11^,^12^,^18^. In this work, we show that the *tgg* single and double mutants show altered leaf epidermal surface and cuticle ultrastructure. We therefore proceeded to investigate if these physical changes were related to clear biochemical differences in the leaves. Through metabolic platforms, we observed differntial levels of fatty acids, indole compounds, glucosinolates and flavonoids in *tgg* single and double mutants, thereby linking chemical and physical defence.

## Results

### *tgg* mutants show altered leaf epidermal surface and cuticle ultrastructure

**Figure 1:**
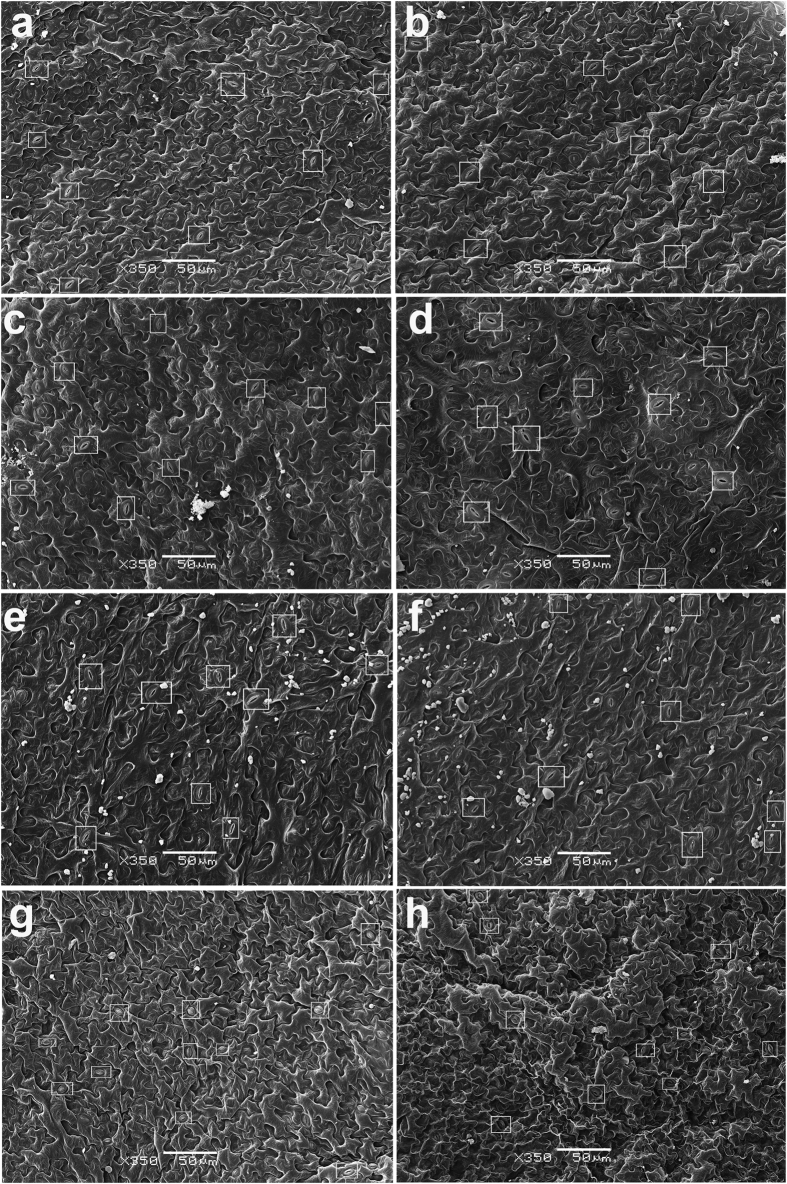
Scanning electron micrographs of abaxial leaf surface of WT, and *tgg1, tgg2* single and *tgg1 tgg2* double mutants of *Arabidopsis*. (**a**,**b**) WT: the non-stomatal pavement cells showing the characteristic jigsaw puzzle shape. (**c**,**d**) *tgg1* single mutant: the pavement cells appeared relatively bigger, but showed jigsaw puzzle shape, and the stomatal guard cells appeared bigger (marked by white rectangles). (**e**,**f**) *tgg2* single mutant: the pavement cells appeared bigger and flattened, showing irregular jigsaw puzzle shape, and the stomatal guard cells also appeared bigger (marked by white rectangles). (**g**,**h**) *tgg1 tgg2* double mutant: the pavement cells appeared overlapping each other, collapsed at some places, and showed irregular jigsaw puzzle shape, and the stomatal guard cells appeared smaller, closed and sunken (marked by white rectangles). (**a–h**) (Scale bars, 50 μm).

**Figure 2:**
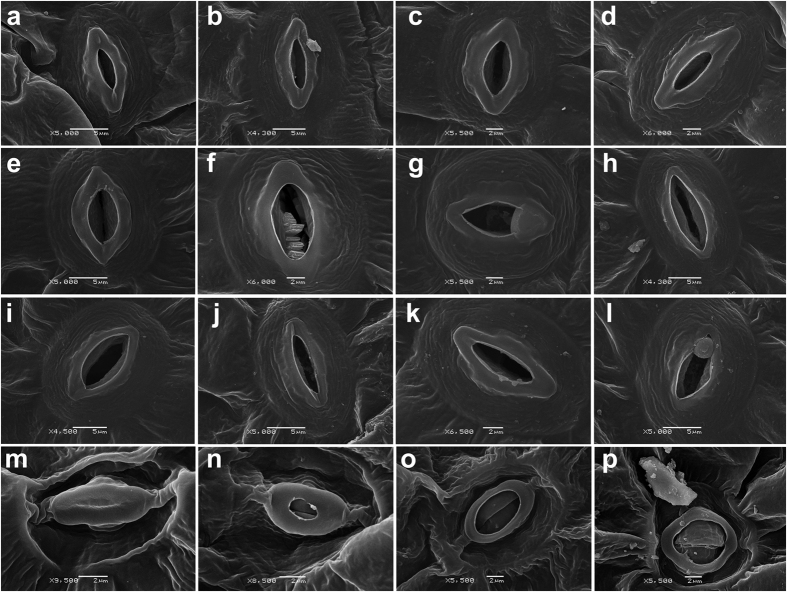
Scanning electron micrographs of stomatal guard cells of WT and *tgg1 tgg2* double mutant. (**a–d**) WT. (**e–h**) *tgg1* single mutant: the stomatal guard cells appear bigger. **(i-l)**, *tgg2* single mutant: the stomatal guard cells appear bigger. (**m–p**) *tgg1 tgg2* double mutant: stomatal guard cells appear smaller, tightly closed and sunken showing variations as compared to the normal and open stomatal guard cells in the WT, and *tgg1* and *tgg2* single mutants. (**a**,**b**,**e**,**h–j**,**l**) (Scale bars, 5 μm). (**c**,**d**,**f**,**g**,**k**,**m–p**) (Scale bars, 2 μm).

**Figure 3:**
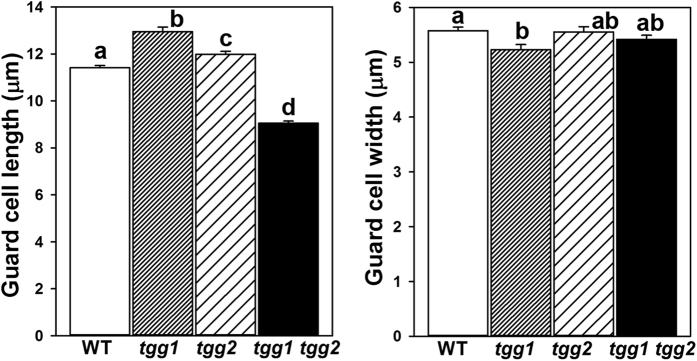
Measures of stomatal size in WT, *tgg1, tgg2* single mutants, and *tgg1 tgg2* double mutant. Average guard cell length and average guard cell width (stomatal aperture) were used as measures of stomatal size. Different letters above the bars indicate significant differences between WT, *tgg1, tgg2* single mutants and *tgg1 tgg2* double mutant (Kruskal-Wallis test, *P* < 0.05), followed by pairwise Wilcoxon Mann-Whitney tests and Bonferroni correction, *P* < 0.00417). Error bars represent the means ± SE (n = 100).

No macroscopic growth/morphological differences were observed between the wild-type (WT) and *tgg* single and double mutants during during the four weeks of plant cultivation (Supplementary Fig. 1, and as reported earlier)^16^. To determine if the WT and *tgg* mutants differ in their leaf epidermal surface, we analyzed the abaxial leaf surfaces of WT and *tgg* single and double mutants using scanning electron microscopy (SEM). Since the abaxial surface of leaf has higher stomatal density and abaxial guard cells are typically larger, we considered to analyse the abaxial surface for SEM analysis^29^,^30^. The SEM images of the abaxial side showed clear differences between the WT and *tgg* mutants for pavement cells, stomata and the presence of wax crystals (Fig. 1). In WT, the pavement cells showed the characteristic jigsaw puzzle shape (Fig. 1a and b). In the *tgg1* single mutant, the pavement cells were bigger in size, but still showing a regular jigsaw puzzle shape as in the WT (Fig. 1c and d). The stomata in the *tgg1* single mutant also appeared bigger (Fig. 2e–h and Fig. 3). In the *tgg2* single mutant, the pavement cells appeared bigger, flattened and showed an irregular jigsaw puzzle shape (Fig. 1e and f). Stomata in the *tgg2* single mutant were also relatively bigger than the WT, and the stomatal aperture was mostly fully open (Figs 2i–l and 3). The pavement cells in the *tgg1 tgg2* double mutant appeared deformed, overlapping each other, collapsed in some places, and hence showed an irregular jigsaw puzzle shape (Fig. 1g and h). Additionally, in the *tgg1 tgg2* double mutant, smaller, tightly closed and sunken stomata were observed (Fig. 1g and h) (Figs 2m–p and 3). WT, *tgg1, tgg2* single mutants, and *tgg1 tgg2* double mutant differ significantly for guard cell length. However, for guard cell width, only WT and *tgg1* single mutant showed significant differences (Fig. 3). In WT, hardly any wax crystals were observed. However, a relatively higher amount of wax crystals was observed on the leaf surfaces of the *tgg* mutants, in particular for the *tgg2* single mutant (Fig. 1e and f), and the *tgg1 tgg2* double mutant (Figs 1g and h and 2).

**Figure 4:**
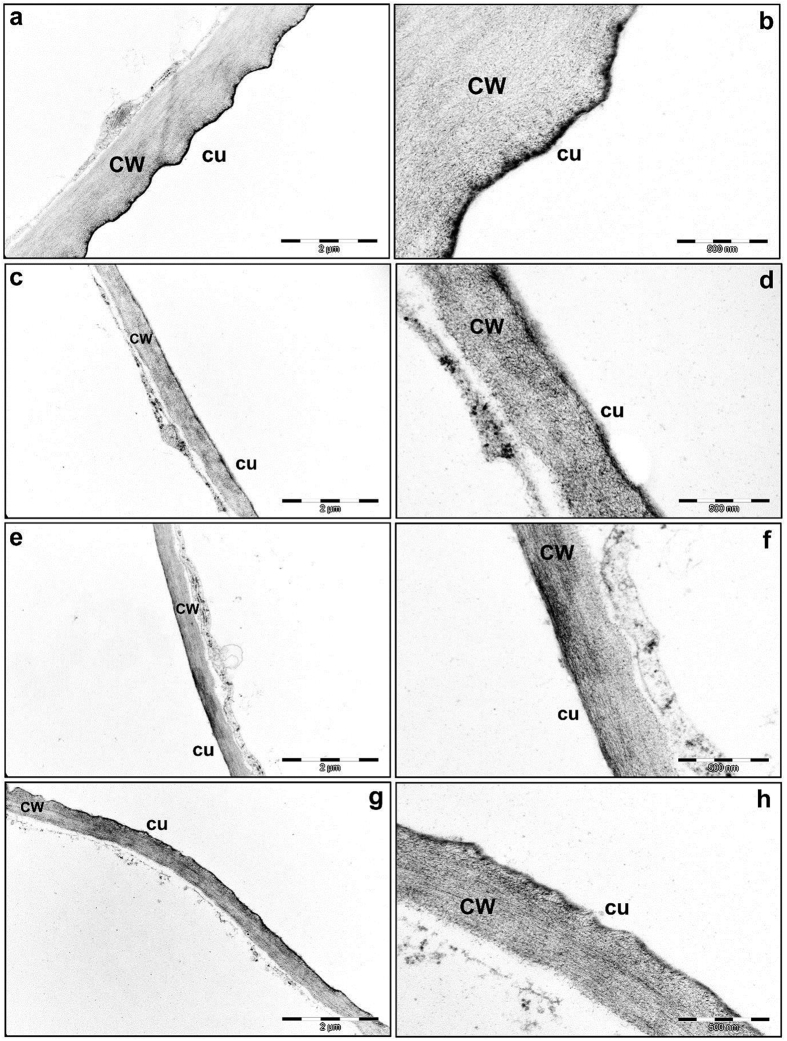
Ultrastructure differences in cuticle of rosette leaves of WT, and *tgg1, tgg2* single and *tgg1 tgg2* double mutants of *Arabidopsis* as examined through transmission electron microscopy (TEM). (**a**,**b**) WT: The cuticle is visible as a continuous, condensed and dark electron dense apposition on the cell wall. (**c**,**d**) *tgg1* single mutant, (**e**,**f**) *tgg2* single mutant, (**g**,**h**) *tgg1 tgg2* double mutant. The cuticle in *tgg1, tgg2* single and *tgg1 tgg2* double mutants is visible as a disrupted and irregular electron-dense apposition on the cell wall. Bars = 2 *μ*m (X 18,500 magnification in **a**,**c**,**e** and **g**), =500 nm (X 68,000 magnification in **b**,**d**,**f** and **h**). Cu, cuticle; and CW, cell wall.

The ultrastructure of the cuticle layer of leaves from WT and *tgg* mutants was analyzed using transmission electron microscopy (TEM). In WT plants, the electron dense layer, representing the cuticle was observed as a regular structure of a condensed and continuous structure outside the cell wall (Fig. 4a and b). However, in both *tgg* single and double mutants the cuticle appeared as disrupted with reduced electron density and appeared to be discontinuous (Fig. 4c–h).

### Compounds detected in WT and *tgg* mutants from fatty acid methyl ester (FAME), cutin and untargeted metabolic profiling analyses

The compounds in both GCMS datasets, fatty acid (FA) and leaf cutin analyses were quantified by comparing their peak intensities to that of the internal standards (Supplementary Figs S1 and S2). Detected compounds, which were annotated on the basis of their retention times, calculated retention indices (RIs), and mass fragments are detailed in (Supplementary Tables S1, S2 and S4).

The untargeted analysis of the GCMS-profiles of the leaf FAME extracts resulted in the detection of 22 FAs, one fatty alcohol (lauryl alcohol), 11 unknown FA phytyl esters (numbered 1–11), and one unknown indole (named indole1) in WT and *tgg1, tgg2* single and *tgg1 tgg2* double mutants (Supplementary Table 1). The quantification of FAs showed 9c12c15c-18:3, 16:0, 7c10c13c-16:3 and 9c12c-18:2 as the most predominant among the 22 FAs detected (Supplementary Fig. 2).

The analysis of the leaf cutin extracts, using GCMS of isolated cuticular waxes, resulted in the detection of 58 compounds, of which 32 represented FAs, nine indoles, nine hydroxycinnamic acids/phenolic compounds, two monoglycerides, one-fatty alcohol, aldehyde, polyol, fatty acid ester, carbohydrate, and diterpene alcohol. (Supplementary Table S2). Among the FAs and indoles, seven FAs and three indoles could not be characterised more accurately from the spectra, which we got, and these have thus been designated as ‘unknown FA’ 1–7 and ‘unknown indole’ 1–3 (mass spectra are provided in Supplementary Table 2). The quantification of FAs acquired from cuticle analysis showed the FAs 16:0, 18:2-diacid, 9c-18:1 and 9c12c-18:2 as being the most predominant FAs among the 32 that were detected (Supplementary Fig. 3).

From LCMS profiling of semi-polar extracts, we identified 18 secondary metabolites in WT and *tgg* mutants, including glucosinolates, flavonoids and phenylpropanoids (Supplementary Table 3).

### Metabolite dependent separation between the wild-type and *tgg* mutants

**Figure 5:**
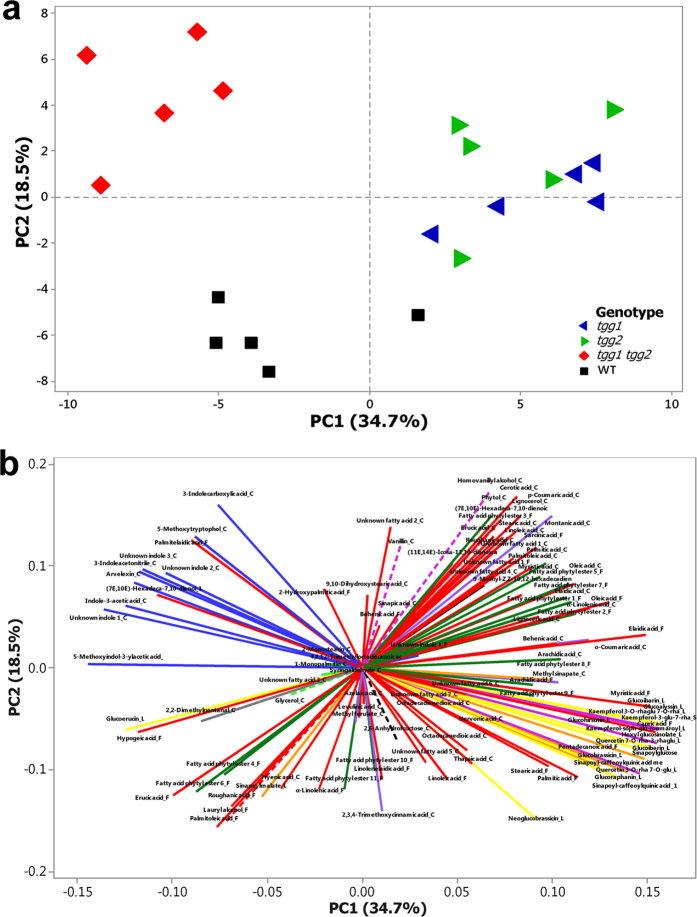
2D principal component analysis (PCA) of 111 compounds (obtained from FAME (F), leaf cutin (C) and LCMS profiling (L) of WT and> *tgg1, tgg2* single and *tgg1 tgg2* double mutants of *Arabidopsis* (n = 5), based on log_2_ ratio amended concentration levels (median) for single metabolites (Supplementary Table S4). A total of 53.2% of variation is explained by PC1 and PC2. (**a**) PCA plot showing differences among wild-type and *tgg* single and double mutants for metabolic profiles. (**b**) Loading plot of metabolites explaining the observed variation in PCA plot, indicated by coloured lines for the different compound groups following the colour scheme used in Fig. 6. 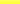 glucosinolates; 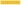 sinapoyl esters; 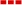 fatty alcohol; 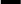 fatty acid ester; 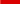 FAs; 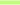 monoglycerides; 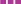 phenolics; 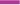 flavonol glycosides; 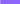 hydroxycinnamic acids; 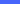 indole compounds; 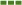 diterpene alcohol; 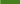 fatty acid phytyl esters; 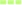 polyols; 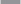 aldehydes; 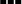 carbohydrate.

**Figure 6:**
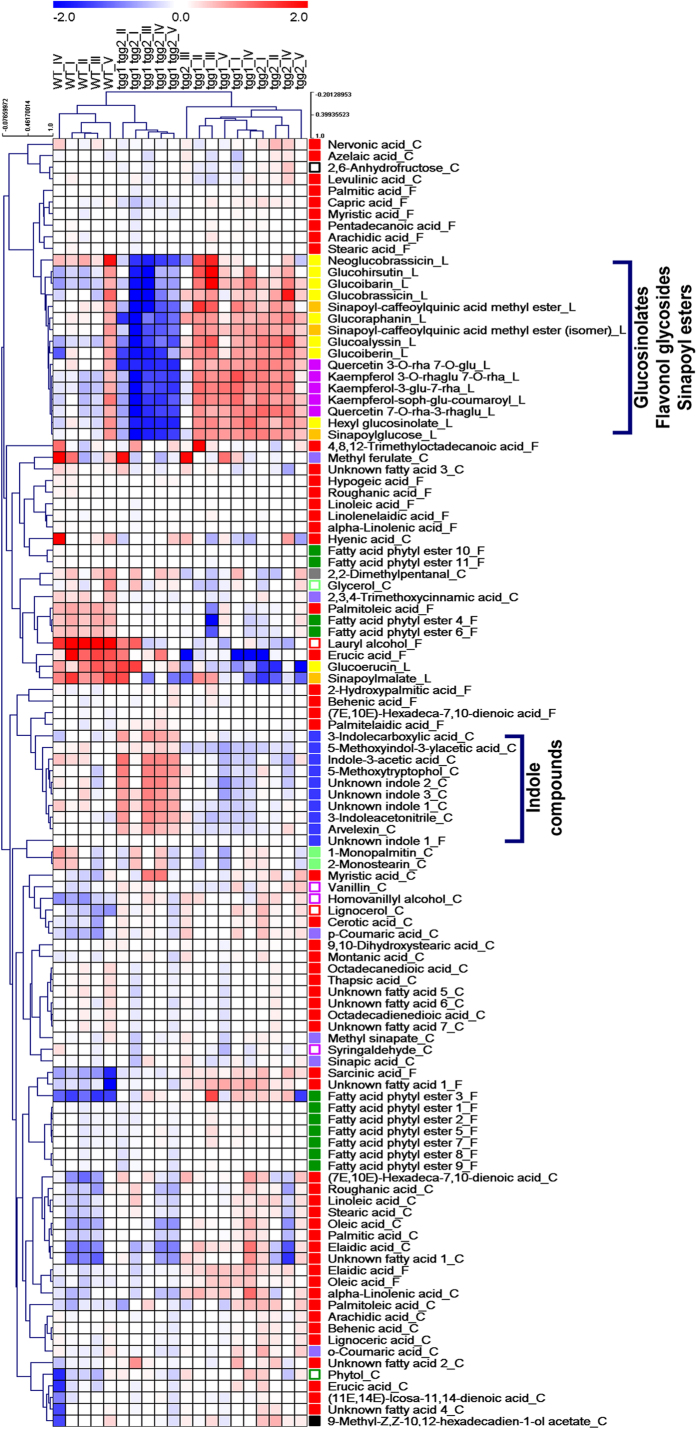
Hierarchical cluster analysis (HCA) (Pearson correlation) of 111 compounds (obtained from FAME (F), leaf cutin (C) and LCMS profiling (L)) of WT and *tgg1, tgg2* single and *tgg1 tgg2* double mutants of *Arabidopsis*. Heat map visualization is based on log_2_(n) ratio amended concentration levels (median) for single metabolites. Bluish colours indicate lower concentration levels, and reddish colours enhanced metabolite levels (see colour scale). Compound group colour code: 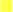 glucosinolates; 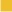 sinapoyl esters; 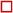 fatty alcohol; 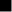 fatty acid ester; 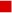 FAs; 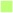 monoglycerides; 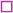 phenolics; 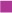 flavonol glycosides; 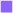 hydroxycinnamic acids; 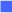 indole compounds; 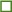 diterpene alcohol; 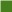 fatty acid phytyl esters; 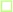 polyols; 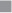 aldehydes; 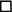 carbohydrate.

Principal component analysis (PCA) was carried out on 111 structurally-annotated/identified metabolites (FAME extracts, leaf cutin monomers and LCMS metabolites including glucosinolates (Supplementary Table S4), which were detected in WT plants, single and/or double mutants. PC1 explained 34.7% of the total variation and coincided with the segregation of WT and the *tgg1 tgg2* double mutant, both of which were separated from the single mutants (Fig. 5a). PC2, explaining 18.5% of the total variation, corresponded to the separation of the double mutant from the WT. The loading plot (Fig. 5b) indicates those metabolites that were mainly responsible for the genotypic separation as shown in Fig. 5a. Segregation of the *tgg1 tgg2* double mutant was mainly due to higher levels of indole compounds (Figs 5b and 6), as observed in the leaf cutin analysis. The clustering of *tgg1* and *tgg2* single mutants (Fig. 5a) was determined by higher levels of metabolite groups comprising glucosinolates, flavonol glycosides and sinapoyl esters in particularly, in addition to certain FAs (Figs 5b and 6).

Hierarchical cluster analysis (HCA) revealed similar cluster patterns for the *tgg1* and *tgg2* single mutants (Fig. 6), which again, were separated from the WT and the *tgg1 tgg2* double mutant. HCA also indicated that both single mutants contained higher levels of aliphatic and indolic glucosinolates, flavonoids and sinapoyl derivatives. In contrast, the levels of these compounds were clearly lower in the *tgg1 tgg2* double mutant, while the levels of indole compounds were enhanced (Fig. 6). The WT plants were characterized by a distinct cluster of various metabolites showing higher levels compared to the mutant plants, including lipid-related compounds (erucic and palmitoleic acid, lauryl alcohol (1-dodecanol), and FA phytyl esters 4 and 6), and secondary metabolites (glucoerucin, sinapoylmalate, neoglucobrassicin and 2,3,4 trimethoxycinnamic acid) (Figs 5b and 6).

### *tgg* mutations alter levels of glucosinolates

**Figure 7:**
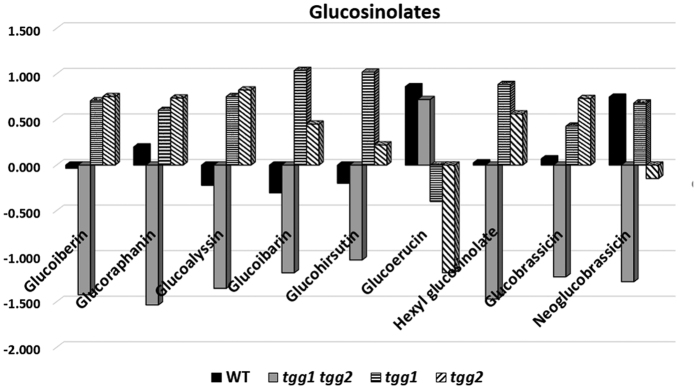
Metabolic levels (log_2_ ratio) of glucosinolates obtained from LC-TOF–MS analyses of WT and *tgg* single and double mutants.

Among the nine glucosinolates detected, eight showed reduced levels in the *tgg1 tgg2* double mutant (Fig. 7). Glucoerucin, was the only glucosinolate that showed higher levels in *tgg1 tgg2* double mutant compared to the *tgg1* and *tgg2* single mutants. However, *tgg1* and *tgg2* single mutants showed moderate to high levels for glucosinolates glucoiberin, glucoraphanin, glucoalyssin, glucoibarin, glucohirsutin, hexyl glucosinolate, and glucobrassicin as compared to the WT (Fig. 7).

### *tgg* mutations affect FAs double bond indices (DBIs)

**Figure 8:**
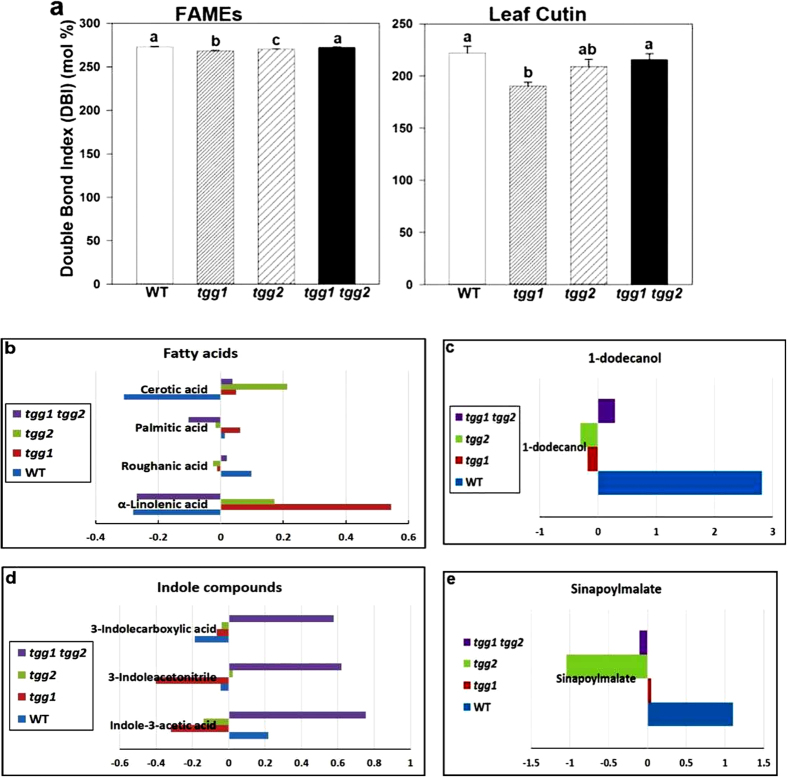
Double bond index (DBI) of fatty acids obtained from FAME and leaf cutin analysis; and metabolic levels (log_2_ ratio) of selected compounds obtained from FAME, leaf cutin and LC-TOF–MS analyses of WT and *tgg* single and double mutants. (**a**) DBI of fatty acids. (**b**) Metabolic levels of some of the differentially accumulated fatty acids between the WT and *tgg* mutants (*P* < 0.05). (**c**) Metabolic levels of differentially accumulated fatty alcohol, 1-dodecanol between the WT and *tgg* mutants (*P* < 0.05). (**d**) Metabolic levels of some of the differentially accumulated indole compounds between the WT and *tgg* mutants (*P* < 0.05), (**e**) Metabolic levels of differentially accumulated compound sinapoylmalate between the WT and *tgg* mutants (*P* < 0.05).

The DBIs were calculated for FAs acquired from fatty acid methyl esters (FAMEs) and leaf cutin analyses based on the intensiy data. The univariate analysis of DBIs for FAs from both analyses showed single mutant *tgg1* to have significantly lower DBIs than the WT (Fig. 8a). The DBIs for FAs, which were acquired from FA-acetyl esters (FAAEs) were slightly lower for the *tgg1 tgg2* double mutant as compared to the WT but did not differ significantly. However, the DBIs for FAs attained from FAME analysis was nearly similar for WT and *tgg1 tgg2* double mutant (Fig. 8a).

## Discussion

The WT and *tgg* single and double mutants did not show any growth/morphological differences during the four weeks of plant cultivation (Supplementary Fig. 1). A similar observation was also made by Barth and Jander^16^. The *tgg1, tgg2* single mutants and *tgg1 tgg2* double mutant plants appeared healthy, viable, grew normally and survived similarly to WT plants. Since we did not observe a clear macroscopic phenotype in *tgg* single or double mutants (Supplementary Fig. 1), we first applied electron microscope techniques to zoom into possible structural alterations in the leaf surface phenotype. The epidermis rosette leaves of both *tgg* single and double mutants showed a lack of the typical jigsaw puzzle shape and a smooth surface, as seen on the abaxial side of WT leaves. This was especially the case for the *tgg1 tgg2* double mutant (Figs 1 and 2). Likewise, the leaf surfaces of *fatty acid desaturase glabra1* (*fad7-1 gl1*) mutants have been shown to exhibit an uneven surface^31^, and curly flag leaf1 (At*CFL1)* overexpressing plants showed crinkled and deformed epidermal cells^32^, indicating effects of fatty acid metabolism on the leaf surface phenotype. *Arabidopsis* wild-type leaves either lack wax crystals^33^,^34^ or have very little very little wax deposition^35^. In agreement with previous observations, we observed only very few and small wax crystals on the abaxial side of leaves of WT plants (Fig. 1). However, small granule-shape wax-like crystals were present on the surface of those of tgg1, *tgg2* single and *tgg1 tgg2* double mutants (Fig. 1). Similar kind of observations of wax deposition on either the abaxial or adaxial sides of surfaces of leaf have been reported for shine (*shns*), *knobhead* (*knb*), *bicentifolia* (*bcf*), *bodyguard* (*bdg*), *lacerata* (*lcr*) and *fiddlehead* (*fdh*) mutants of *Arabidopsis*^35^,^36^,^37^.

Stomata in the *tgg1 tgg2* double mutant were relatively small, closed and shrunken, as compared to WT plants (Figs 1 and 2). A similar observation has been made in *hat1* (*harmattan tolerant*) mutant under mild xerothermic stress^38^: under dry and hot growth conditions the *hat1* stomata crumpled and sank more than the WT ones, resulting in a smaller stomatal aperture and sunken stomata, particularly in combination with soil drought. The *hat1* plants are also ABA-hypersensitive. Our results therefore suggest that *tgg1 tgg2* double mutant plants show characteristics of a plant under drought stress, possibly involving ABA triggering stomatal closure and thereby reducing water loss^38^,^39^,^40^. The stomata with closed aperture in *tgg1 tgg2* double mutant (Figs 1 and 2) fits well with the knowldege that the *TGG1* gene is expressed in stomata guard cells and its expressed protein is strikingly abundant in these cells^4^,^15^. Having observed these structural differences, it was then important to investigate if these were also linked to metabolic perturbations in the leaf itself.

### Metabolite alterations in *tgg* mutants have relevance to stomatal cells, guard cells, and (a)biotic stress responses

TGG1 is an abundant protein in the guard cell proteome^4^ and appears to play an essential role in guard cell ABA signalling. The mutant *tgg1* plants used in the present study lack this enzyme and were unresponsive to ABA-dependent inhibition of stomatal opening and in K^+^-channel regulation^4^. Accordingly, the enhancement in the guard cell proteome of proteins involved in FA biosynthesis may show not only the importance of lipids to cuticle formation in the leaf, but also the potential significance of lipids and lipid metabolites as signalling entities in guard cells^4^,^7^. Our metabolite analyses indicate differential accumulation of several compounds frequently associated with stomata opening and abiotic stress resistance mechanisms. For example, in epidermal peels of *Commelina communis*, α-linolenic acid (9c12c15c-18:3), the levels of which are significantly different among WT and *tgg* mutants) (Fig. 8b) enhanced stomatal opening at sub-saturating levels of white light, while it inhibited darkness-induced stomatal closure^41^. High-temperature acclimatization caused considerable decreases in trienoic FAs roughanic acid (levels significantly different among WT and *tgg* mutants) (Fig. 8b), and 9c12c15c-18:3, with parallel increases in levels of the less unsaturated FAs palmitic acid and linoleic acid^42^. Trienoic FAs are required for low-temperature recovery from photoinhibition and for long-term thermotolerance^43^,^44^. Water deficiency increased the total cutin monomer levels by 65%, while both water shortage and NaCl changed proportionally the levels of cutin monomers^45^. Levels of VLFA cerotic acid (significantly different among WT and *tgg* mutants) (Fig. 8b), was observed to be present in elevated amount in *cer9* wax mutant^46^,^47^. Additionally, the *CER9* gene has been shown to play a key role as a regulator of plant water use efficiency and in overall plant stress responses. This indicated that *CER9* may encode an important new cuticle-associated drought-tolerance determinant protein^47^. Dodecanol (lauryl alcohol), which was relatively low in all *tgg* mutants (Fig. 8c), was increased in *Arabidopsis* leaves infested with *Pieris rapae*^48^, may be involved in the initiation of defence.

### Differential levels of secondary metabolites in *tgg* mutants may have implication in plant defence occurring at the leaf surface and (a)biotic responses

In our previous studies with *Brassica napus* transgenic *MINELESS* plants, which are deficient in myrosinase activity, we showed that *MINELESS* seedlings accumulated higher levels of glucosinolates^14^. Likewise, in the present study, the levels of certain glucosinolates were observed to be different in leaves of WT and *tgg* single and double mutants (Figs 6 and 7). The increased levels of glucosinolates in leaves of *tgg1* and *tgg2* single mutants, compared to the WT plants as reported here, might be explained by a lower degradation resulting from reduced thioglucosidase (myrosinase) gene expression. In contrast, the lower levels of glucosinolates except glucoerucin, in the *tgg1 tgg2* double mutant as compared to the WT plants (Figs 6 and 7), can be possibly due to total lack of myrosinase, leading to negligible production of glucosinolate-myrosinase hydrolysis products, and thus no glucosinolate biosynthesis. Interestingly, we observed relatively high levels of several indole compounds in the cutin fraction from the *tgg1 tgg2* double mutant, as compared to both the single mutants and WT plants, where indole-3-acetic acid, 3-indoleacetonitrile, and 3-indolecarboxylic acid represent some of the important examples (Fig. 8d). The role of indole-derived compounds has also been linked to the establishment and maintenance of plant systemic immunity^49^. Moreover, it has been reported that cell walls of *Arabidopsis* may contain indole compounds and that these cell wall-bound indoles perhaps play a role in pathogen resistance^50^.

Flavonoids, detected mainly as kaempferol and quercetin glycosides, showed higher levels in *tgg1* and *tgg2* single mutants and reduced levels in *tgg1 tgg2* double mutant (Fig. 6). The compound sinapoylmalate, here showing higher abundance in the WT than in *tgg* single and double mutants (Fig. 6 and 8e), is a UV fluorescent secondary metabolite that accumulates in the adaxial leaf epidermis^51^. Kim and coworkers further showed that the *reduced epidermal fluorescence5* (*ref5-1*) mutant with a missense mutation in *CYP83B1* (involved in the production of indole glucosinolates), has reduced sinapoylmalate and indole glucosinolate content^51^. The *cyp83a1* mutants were found to be strongly affected in the accumulation of sinapoylmalate and leaf cuticular wax deposition^49^. Recently, it has been proposed that volatiles must first cross membrane(s), the aqueous environment of the cell wall, and usually also the cuticle, before being emitted into external gas phase^16^. From this opinion paper, we can speculate that the altered levels of cuticle compounds in *tgg* single and double mutants, which have fewer or lower levels of isothiocyanate volatiles^16^, may also impact the biological mechanisms involved in trafficking of other hydrophobic compounds. Since the plant cuticle imposes a significant resistance to volatile organic compound (VOC) emission, another important area of research which needs further attention, is the impact of cuticle composition on VOC emission^52^, especially in the context of biotic resistance.

In conclusion, the changes observed in the different thioglucosidase (*tgg*) mutants investigated here highlight a group of compounds, which are known for their implication in plant defence mechanisms against a/biotic stress. Additionally, these distinct results on differences between WT and *tgg* mutants regarding important bioactive compounds, link the plant chemotype with cuticle structural phenotype. These results therefore provide a foundation for a novel link between chemical, cellular and physical defence systems.

## Methods

### Plant material and growth conditions

Seeds of *Arabidopsis* WT (Col-0), and *tgg1, tgg2* single mutants and *tgg1 tgg2* double mutant lines^16^ were stratified for 2 days at 4 °C and then transferred onto a soil mixture (1:1:1 peat moss-enriched soil/vermiculite/perlite) in 30 mm pots. The plants were grown in a randomized order in growth-room at 22 °C/18 °C, 40/70% relative humidity, a 16/8 h light/dark period, and at 80 μmol m^−2^ s^−1^ light intensity. The leaves from 12 plants at four weeks after planting were pooled in a randomized order to make one biological sample for each of the WT and *tgg* mutants, by snap-freezing rosette leaves in liquid nitrogen. This way, five biological replicates were made for each of the WT and *tgg1, tgg2* single and *tgg1 tgg2* double mutants. The frozen material was ground into a fine powder in liquid nitrogen and the samples were used for the metabolite analyses.

### SEM and TEM analyses

For SEM analysis, leaf tissues from about 3–4 weeks old plants were cut in small pieces (about 2 × 2 mm), and fixed with glutaraldehyde (2%) in Sørensen’s phosphate buffer (0.1 M, pH 7.2) overnight at room temperature. After washing in buffer, the samples were dehydrated through a graded ethanol series at room temperature following the method^53^. After drying in a critical point dryer (Polaron) with liquid CO_2_, the samples were mounted on aluminium stubs for SEM, and then coated with a thin film (~30 nm) of gold-palladium for 3 min by using (2, 5 kV, 20 mA) in a sputter coater (Polaron coating unit E5100). The images were taken with scanning electron microscope JSM-6480 (JEOL). The guard cell length and guard cell width have been measured from several images of WT, *tgg1, tgg2* single mutants and the *tgg1 tgg2* double mutant.

For TEM analysis, following the method^53^, small pieces of leaf tissues were fixed with glutaraldehyde (2.5%) and paraformaldehyde (2%) in Sørensen’s phosphate buffer (0.1 M, pH 7.2) overnight at room temperature. After washing in buffer, the samples were post fixed in 2% osmium tetroxide in Sørensen’s phosphate buffer for 1 h at room temperature. The samples were washed in buffer and dehydrated through a graded ethanol series at room temperature. After infiltration with propylene oxide and epoxy resin LX-112, the samples were embedded in Epoxy resin LX-112, which was polymerised at 57 °C for 3 days. Semi-thin sections (1 μm) were cut with an ultra-microtome (Leica EM UC6) using glass knives, and stained with toluidine blue for orientation and trimming of the blocks. From the areas selected, ultrathin sections (thickness = 70 nm) were cut using a diamond knife, and collected on formvar-coated copper slot grids. The grids were stained with (4% uranyl acetate in 50% ethanol) for 25 min, and alkaline lead citrate (1% in 0.2 M NaOH) for 5 min and then transferred to a grid box. The grids were viewed with a transmission electron microscope JEM 1011 (JEOL), equipped with a digital camera Morada, operating at 80 kV.

### Extraction of FAs, leaf cutin and GCMS analysis

FAs were extracted and analyzed as FAMEs as described by^54^ with minor modifications. In short, 250 mg of leaf material was weighed and extracted with 4 ml of CHCl_3_:MeOH (2:2.5 v/v) with heptadecanoic acid (C17:0, Sigma) as an internal standard. Two extra tubes were made without samples just to have standard heptadecanoic acid (in duplicate). These were started at the step when the internal standards were added to tubes containing samples. Subsequently, these tubes were subjected to the rest of the procedure together with the sample tubes and were used as negative controls. The FAs present in the lipids of the dried chloroform extracts were converted into FAMEs using a 3 ml solution of 5% (v/v) H_2_SO_4_ in methanol. After extraction with hexane, FAMEs were analysed using GCMS, as described previously^55^. The solvent (hexane), standards and sample tubes were run on the GCMS in a single series.

Leaf cutin analysis was performed based on the methods described previously^56^. In short, per sample 750 mg of frozen leaf powder was immersed and homogenized with hot isopropanol (25 ml per g tissue). The insoluble pellet was cleaned by washing twice with chloroform-methanol mixtures to remove free lipid soluble compounds, and the residue dried and subsequently subjected to methanolysis with sodium methoxide and acetylation with acetic anhydride in pyridine. The residue, dried to constant weight, was heated at 60 °C with periodic vortexing in MeOH containing 15% (v/v) methyl acetate and 6% (w/v) sodium methoxide. Heptadecanoic acid (17:0), Sigma, and pentadecanolactone (Aldrich) were added as internal standards at 1 mg g^−1^ to dried residue. Four extra tubes were made without samples just to have standard heptadecanoic acid (in duplicate) and standard pentadecanolactone (in duplicate). This was started at the same step when the internal standard were added to sample containing tubes, and then rest of the procedure was followed similarly for samples and without samples (with standards only). These tubes were used as negative controls. The monoesters (FA-acetyl esters; FAAEs) were finally dissolved in heptane: toluene (1:1; v/v) before analysis by GCMS. The solvent heptane: toluene (1:1; v/v), standards and sample tubes were run on the GCMS in a single series.

Both FAME extracts (organic solvent-soluble methylated compounds) and FAAEs (organic solvent-insoluble acetylated compounds) were analysed on an Agilent model 7890 gas chromatograph using a DB-225 column (Agilent technologies; 30 m × 250 μm internal diameter and 0.25 μm film thickness) and ChemStation software (Agilent Technologies) to acquire data. The data was imported into Excel, and processed as described under data processing, identification and annotation of compounds.

### LC-TOF–MS analysis for untargeted profiling of semi-polar metabolites

Semi-polar leaf compounds were extracted with aqueous-methanol and analysed by liquid chromatography coupled to a photodiode array detector and a quadrupole time of flight high-resolution mass spectrometry (LC-PDA-QTOF-MS) system, using C18-reversed phase chromatography and negative electrospray ionization, as described previously^57^. For metabolite extraction, 100 mg of fine-powdered leaf material was mixed with 750 μl of ice-cold 86% methanol acidified with 0.114% (v/v) formic acid. After vortexing for 5 s, sonication for 15 min and centrifugation (12,000 rpm) for 10 min, the extracts were filtered through syringe filters (Minisart SRP 4, 0.45 μm, Sartorius Stedim Biotech), and collected in glass vials.

### Data processing, identification and annotation of compounds

Both GCMS and LCMS data sets were processed using the MetAlign software package (www.metalign.nl) for baseline correction, noise estimation, and ion-wise mass alignment^58^. The in-house script, MetAlign Output Transformer^59^ was used for filtering out low and irreproducible signals and noise value imputation. The resulting mass peak lists were subjected to MSClust^60^ to group the mass signals originating from the same compound based on their similar retention time and intensity patterns across samples.

The putative identification of GCMS compounds was performed through the matching of their mass spectra extracted by MSClust to the National Institute of Standards and Technology (NIST) mass spectral library entries, using the NIST MS Search v2.0 software tool (http://chemdata.nist.gov/mass-spc/ms-search/), and to an in-house mass spectral database for GCMS. Both mass spectra and retention indices of the reconstructed metabolites were used to search for putative candidate metabolites already reported for *Arabidopsis*. The annotation of the predominant FAs was verified using authentic standards. RIs were calculated using a standard mixture of *n*-alkanes and FAMEs, in combination with published RIs of FAMEs (e.g., NIST Standard Reference Database). The identification of metabolites acquired from LCMS analysis was performed by means of their UV spectra, the exact molecular weight, by using in-house metabolite databases and as earlier described^61^,^62^.

### Double bond index

The double bond index (DBI) was calculated for FAs attained from FAME extracts and FAAEs based on the intensity data (detector response) as follows:


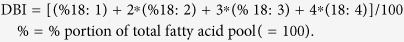


### Data analysis

Multivariate data analyses were based on 111 identified or structurally-annotated metabolites (Supplementary Tables S1,S2,S3,S4), which were obtained from GCMS-based FAME and cuticle, and LCMS-based untargeted metabolic profiling. Principal component analysis (PCA) was carried out using Minitab^®^ software (v.17.1.0; Minitab, Ltd., Coventry, UK) (Fig. 5). Hierarchical clustering analysis (HCA) based on Pearson correlation was achieved with MultiExperiment Viewer software v. 4.9.0 (http://www.tm4.org/mev.html), with distinct compound groups being highlighted by a colour code (Fig. 6) (Supplementary Table S4).

The statistical analysis on 111 identified or structurally-annotated metabolites quantified FAs (Supplementary Tables S1,S2,S3,S4) (Supplementary Figs S1 and S2) was performed using the software package R^63^. Normality assumption was checked using quantile-quantile-plots and the Anderson-Darling test. The results showed that the normality assumption in general was not satisfied, and therefore non-parametric tests were used for analysis. The datasets were analysed using the Kruskal Wallis test, which is the non-parametric alternative to one-way ANOVA. For the Kruskal Wallis test, *P* < 0.05 was considered significant. The compounds showing significant results using Kruskal-Wallis test were further analysed by pairwise comparisons of subgroups using Wilcoxon Mann-Whitney test. For the pairwise comparisons, we used the Benjamini-Hochberg procedure to correct for multiple testing, by calculating adjusted *P* values. An adjusted *P* value (*P* < 0.05) is considered significant. Five observations were available in each group and the multcompView R-package (version 0.1–7) was used to categorize the four groups based on significance, as presented in (Figs S2 and S3), (http://cran.r-project.org/ and choose package multcompView). The statistical analysis on DBI of FAs attained from FAME extracts and FAAEs was performed using two-way ANOVA coupled with Tukey’s test (*P* value < 0.05).

The data for both guard cell length and guard cell width (stomtatal aperture) were analyzed using Kruskal-Wallis one-way ANOVA. Multiple testing correction was done using the Bonferroni correction, which means (*P* value < 0.025) were considered significant. Tweleve pairwise comparisons (six for guard cell length, and six for guard cell width) among subgroups were done using Wilcoxon Mann-Whitney test. Multiple testing correction for the Wilcoxon Mann-Whitney test was done using the Bonferroni correction, which means *P* values < 0.00417 were considered significant.

## Additional Information

**How to cite this article**: Ahuja, I. *et al*. *Arabidopsis* myrosinases link the glucosinolate-myrosinase system and the cuticle. *Sci. Rep.* **6**, 38990; doi: 10.1038/srep38990 (2016).

**Publisher's note:** Springer Nature remains neutral with regard to jurisdictional claims in published maps and institutional affiliations.

## Supplementary Material

Supplementary Information

Supplementary Table S4
