# Supplementary material for: Arabidopsis myrosinases link the glucosinolate-myrosinase system and the cuticle
Source: Sci Rep. 2016 Dec 15;6:38990. doi: 10.1038/srep38990 (PMC5157024; doi:10.1038/srep38990)
Supplement: Supplementary Information [file srep38990-s1.pdf]

Arabidopsis myrosinases link the glucosinolate-myrosinase system and the cuticle

Ishita Ahuja[1,2,3], Ric C. H. de Vos[2†], Jens Rohloff[1†], Geert Stoopen[2], Kari K. Halle[4], Samina Jam Nazeer Ahmad[5], Linh Hoang[6], Robert D. Hall[2, 7, 8†], Atle M. Bones[1†*]

1. Department of Biology, Norwegian University of Science and Technology (NTNU), Realfagbygget, NO-7491 Trondheim, Norway
2. Plant Research International, P.O. Box 16, 6700 AA Wageningen, The Netherlands
3. Norwegian Institute for Nature Research (NINA), NO-7485 Trondheim, Norway
4. Department of Mathematical Sciences, NTNU, Trondheim, Norway.
5. Department of Botany, University of Agriculture Faisalabad (UAF), Pakistan
6. Cellular and Molecular Imaging Core Facility (CMIC), Laboratory for Electron Microscopy, NTNU, Trondheim, Norway
7. Laboratory of Plant Physiology, Wageningen University, P.O. Box 16, 6700 AA Wageningen, The Netherlands
8. Netherlands Metabolomics Centre, Einsteinweg 55, 2333 CC Leiden, The Netherlands

[†] These senior authors contributed equally.

* To whom correspondence should be addressed.
E-mail: atle.m.bones@ntnu.no
Phone: +47-73-898692
Fax: +47-73-596100

Date of submission: 25/10/16

Figure S1. Morphological appearance of 3-4 weeks old plants of wild-type and *tgg* single and double mutants. (Scale bar = 1 cm).

Figure S2. The contents of FAs (nmol g-1 tissue wt.) (Fresh wt. basis) in rosette leaves of wild-type, *tgg1*, *tgg2* single and *tgg1 tgg2* double mutants from FAME analysis. Error bars represent S.E.

Figure S3. The contents of FAs in rosette leaves of wild-type, *tgg1*, *tgg2* single and *tgg1 tgg2* double mutants from leaf cutin analysis. Error bars represent S.E.

Table S1. Compounds detected in leaves of wild-type, *tggl, tgg2* single and *tggl tgg2* double mutants of *Arabidopsis* from FAME analysis.

Table S2. Compounds detected in wild-type, *tggl, tgg2* single and *tggl tgg2* double mutants of *Arabidopsis* from leaf cutin analysis.

Table S3. Compounds detected by LC-QTOF-MS (ESI negative mode) in leaves of wild-type, *tggl, tgg2* single and *tggl tgg2* double mutants of *Arabidopsis* through untargeted metabolic profiling.

Table S4a. The list of structurally annotated/identified metabolites (obtained from FAME (F), leaf cutin (C) and untargeted metabolic profiling (L)) with values (log2 ratio) used for 2D principal component analysis (PCA) and Hierarchical cluster analysis (HCA).

Table S4b. The list of structurally annotated/identified metabolites (obtained from FAME (F), leaf cutin (C) and untargeted metabolic profiling (L)) with peak intensity values which were used to quantify log2 ratios (Table S4a).

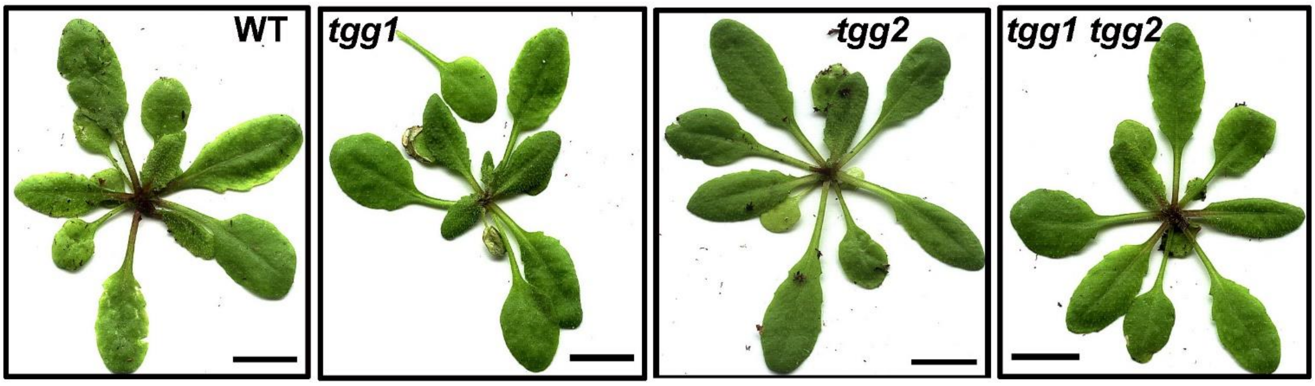

Figure S1

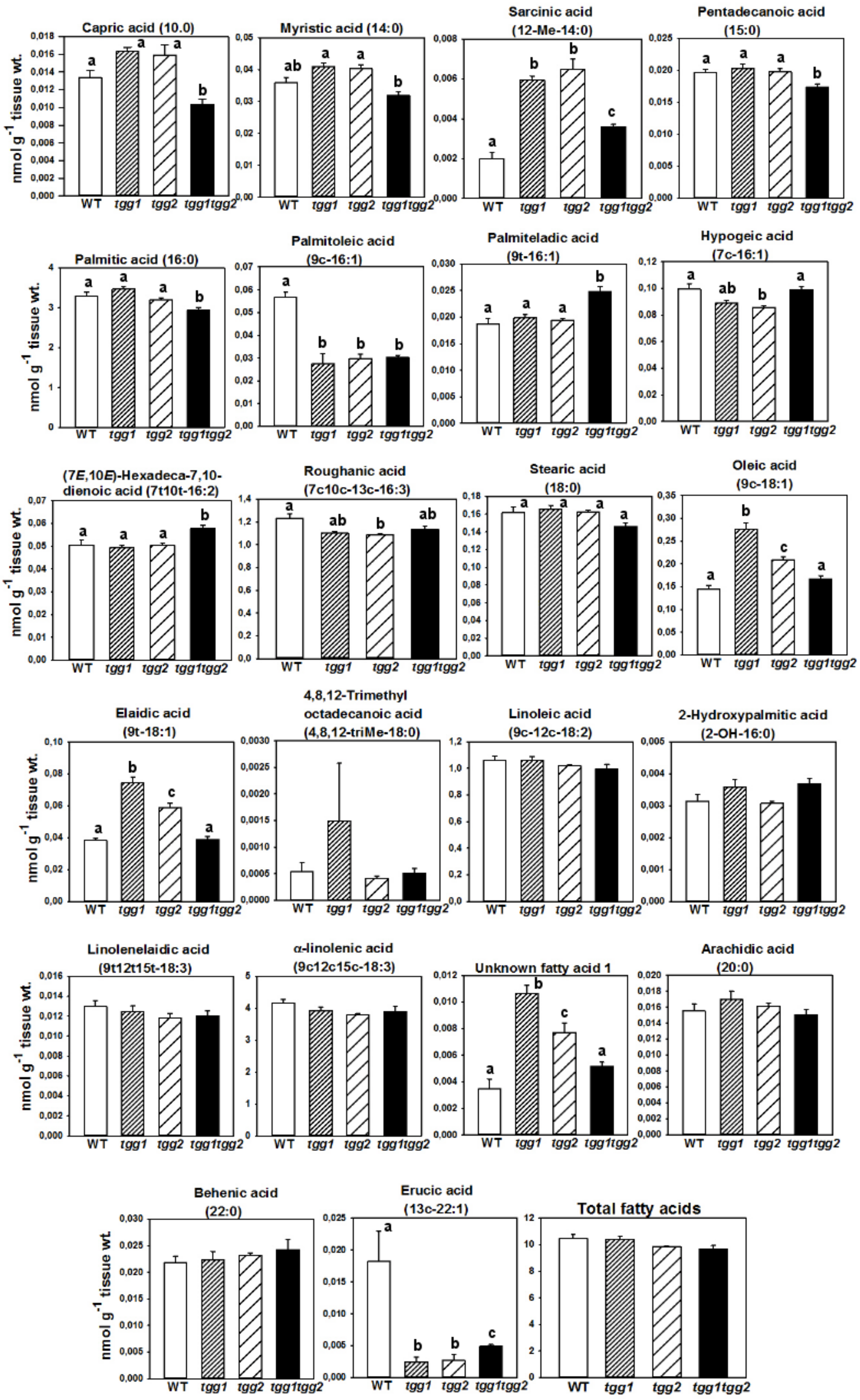

Capric acid (10.0)
Myristic acid (14:0)
Sarcinic acid
(12-Me-14:0)
Pentadecanoic acid
(15:0)
Palmitic acid (16:0)
Palmitoleic acid
(9c-16:1)
Palmiteladic acid
(9t-16:1)
Hypogeic acid
(7c-16:1)
(7E,10E)-Hexadeca-7,10-
dienoic acid (7t10t-16:2)
Roughanic acid
(7c10c-13c-16:3)
Stearic acid
(18:0)
Oleic acid
(9c-18:1)
Elaidic acid
(9t-18:1)
4,8,12-Trimethyl
octadecanoic acid
(4,8,12-triMe-18:0)
Linoleic acid
(9c-12c-18:2)
2-Hydroxypalmitic acid
(2-OH-16:0)
Linolenelaidic acid
(9t12t15t-18:3)
α-linolenic acid
(9c12c15c-18:3)
Unknown fatty acid 1
Arachidic acid
(20:0)
Behenic acid
(22:0)
Erucic acid
(13c-22:1)
Total fatty acids
nmol g -1 tissue wt.
WT
tgg1
tgg2
tgg1tgg2

Figure S2

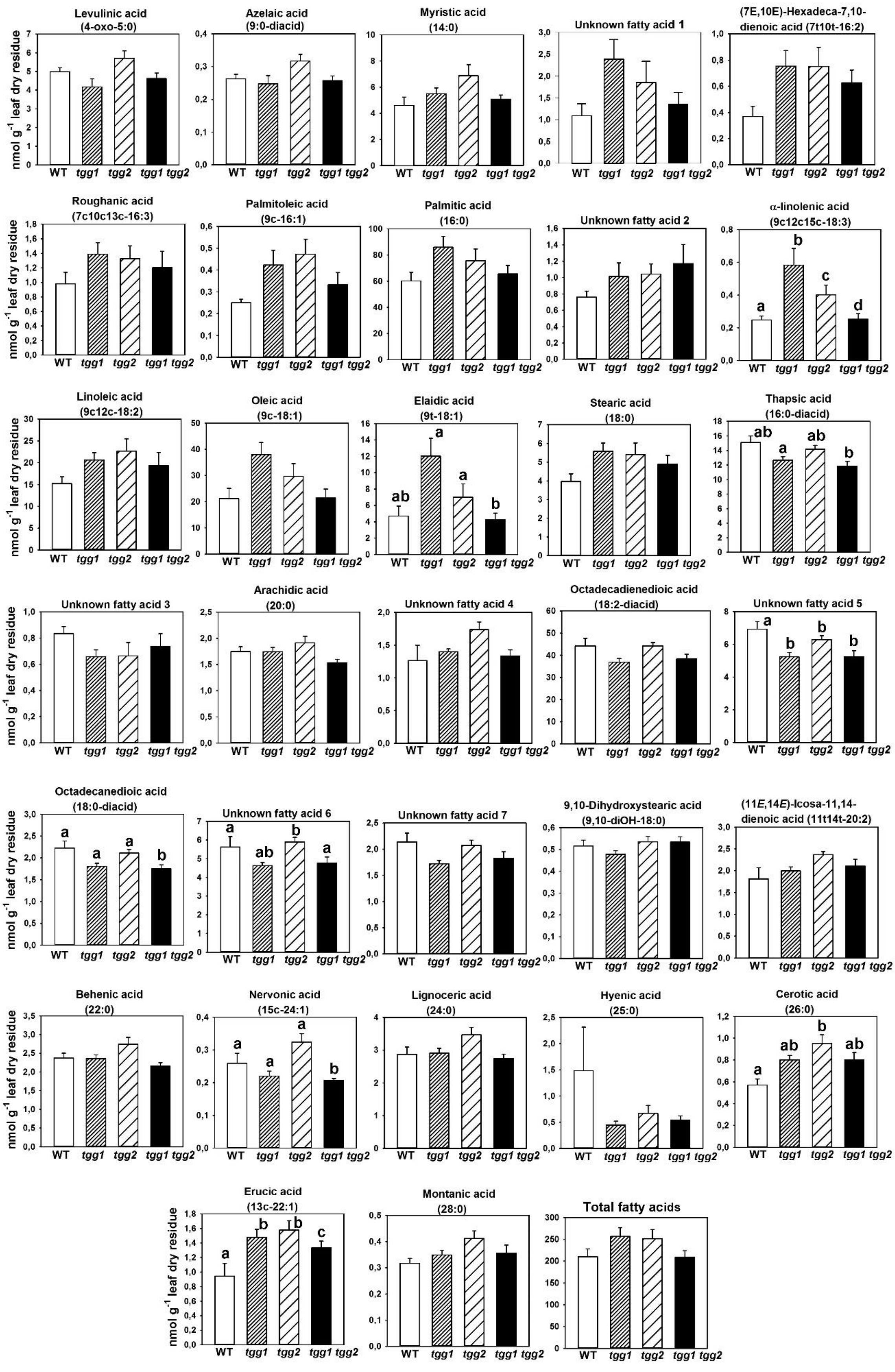
Levulinic acid (4-oxo-5:0)
Azelaic acid (9:0-diacid)
Myristic acid (14:0)
Unknown fatty acid 1
(7E,10E)-Hexadeca-7,10-dienoic acid (7t10t-16:2)
Roughanic acid (7c10c13c-16:3)
Palmitoleic acid (9c-16:1)
Palmitic acid (16:0)
Unknown fatty acid 2
α-linolenic acid (9c12c15c-18:3)
Linoleic acid (9c12c-18:2)
Oleic acid (9c-18:1)
Elaidic acid (9t-18:1)
Stearic acid (18:0)
Thapsic acid (16:0-diacid)
Unknown fatty acid 3
Arachidic acid (20:0)
Unknown fatty acid 4
Octadecadienedioic acid (18:2-diacid)
Unknown fatty acid 5
Octadecanedioic acid (18:0-diacid)
Unknown fatty acid 6
Unknown fatty acid 7
9,10-Dihydroxystearic acid (9,10-diOH-18:0)
(11E,14E)-Icosa-11,14-dienoic acid (11t14t-20:2)
Behenic acid (22:0)
Nervonic acid (15c-24:1)
Lignoceric acid (24:0)
Hyenic acid (25:0)
Cerotic acid (26:0)
Erucic acid (13c-22:1)
Montanic acid (28:0)
Total fatty acids
nmol g-1 leaf dry residue
WT tgg1 tgg2 tgg1 tgg2

Figure S3

Table S1. Compounds detected in leaves of wild-type, *tgg1*, *tgg2* single and *tgg1 tgg2* double mutants of *Arabidopsis* from FAME analysis.

**Compounds with systematic and common names**	**Structure/MS unknowns**	**Molecular structure**	**Retention Index (RI)**
**Fatty acyls**			
Decanoic acid (Capric acid)	10:0	$C_{10}H_{20}O_2$	1325
Tetradecanoic acid (Myristic acid)	14:0	$C_{14}H_{28}O_2$	1704
12-Methyltetradecanoic acid (Sarcinic acid)	12-Me-14:0	$C_{15}H_{30}O_2$	1756
Pentadecanoic acid	15:0	$C_{15}H_{30}O_2$	1794
Hexadecanoic acid (Palmitic acid)	16:0	$C_{16}H_{32}O_2$	1894
(Z)-9-Hexadecenoic acid (Palmitoleic acid)	9c-16:1	$C_{16}H_{30}O_2$	1904
(E)-9-Hexadecenoic acid (Palmitelaidic acid)	9t-16:1	$C_{16}H_{30}O_2$	1905
(Z)-7-Hexadecenoic acid (Hypogeic acid)	7c-16:1	$C_{16}H_{30}O_2$	1920
(7E,10E)-Hexadeca-7,10-dienoic acid	7t10t-16:2	$C_{16}H_{28}O_2$	1939
(7Z, 10Z,13Z)-Hexadeca-7,10,13-trienoic acid (Roughanic acid)	7c10c13c-16:3	$C_{16}H_{26}O_2$	1987
Octadecanoic acid (Stearic acid)	18:0	$C_{18}H_{36}O_2$	2103
(Z)-9-Octadecenoic acid (Oleic acid)	9c-18:1	$C_{18}H_{34}O_2$	2120
(E)-9-Octadecenoic acid (Elaidic acid)	9t-18:1	$C_{18}H_{34}O_2$	2126
4,8,12-Trimethyloctadecanoic acid	4,8,12-triMe-18:0	$C_{21}H_{42}O_2$	2136
(9Z,12Z)-Octadeca- 9,12-dienoic acid (Linoleic acid)	9c12c-18:2	$C_{18}H_{32}O_2$	2158
2-Hydroxyhexadecanoic acid (2-Hydroxypalmitic acid)	2-OH-16:0	$C_{16}H_{32}O_3$	2187
(9E,12E,15E)-Octadeca-9,12,15-trienoic acid (Linolenelaidic acid)	9t12t15t-18:3	$C_{18}H_{30}O_2$	2197
(9Z,12Z,15Z)-Octadeca-9,12,15-trienoic acid (α-Linolenic acid)	9c12c15c-18:3	$C_{18}H_{30}O_2$	2213
Unknown fatty acid 1	79(100), 91(77), 67(59), 55(37), 105(25)	-	2229
Icosanoic acid (Arachidic acid)	20:0	$C_{20}H_{40}O_2$	2323
Docosanoic acid (Behenic acid)	22:0	$C_{22}H_{44}O_2$	2538
(Z)-13-Docosenoic acid (Erucic acid)	13c-22:1	$C_{22}H_{42}O_2$	2558
**Fatty alcohol**			
1-Dodecanol (Lauryl alcohol)	1-OH-12	$C_{12}H_{26}O$	1472
**Fatty acid phytyl esters**			
Fatty acid phytyl ester 1	123(100), 81(39), 95(21), 55(14), 278(12)	-	1633
Fatty acid phytyl ester 2	123(100), 81(35), 95(18), 55(14), 278(11)	-	1636
Fatty acid phytyl ester 3	95(100), 81(74), 123(61), 69(43), 57(31)	-	1646
Fatty acid phytyl ester 4	68(100), 95(89), 82(75), 57(45), 81(42), 55(41), 123(40)		1656
Fatty acid phytyl ester 5	123(100), 81(32),	-	1662

	95(17), 55(13), 278(12)		
Fatty acid phytyl ester 6	81(100), 95(90), 68(79), 57(62), 123(61), 55(13), 278(14)		1681
Fatty acid phytyl ester 7	123(100), 81(32), 95(16), 55(12), 278(12)	-	1688
Fatty acid phytyl ester 8	82(100), 95(59), 57(27), 67(23), 123(21), 81(21), 278(14)		1701
Fatty acid phytyl ester 9	82(100), 95(74), 57(27), 67(23), 123(22), 81(22), 278(17)		1723
Fatty acid phytyl ester 10	81(100), 123(36), 69(19), 85(19), 57(16), 278(4)		1849
Fatty acid phytyl ester 11	81(100), 123(30), 85(23), 57(20), 95(20), 69(17), 278(5)		1851
**Indoles**			
Indole 1	99(100), 191(80), 57(13), 206(10), 163(7)	-	1350

Table S2. Compounds detected in wild-type, *tggl*, *tgg2* single and *tggl tgg2* double mutants of *Arabidopsis* from leaf cutin analysis.

Compounds with systematic and common names	Structure/ MS unknowns	Molecular structure	Retention Index (RI) (analyte)
**Fatty alcohol**			
Tetracosanol (Lignocerol)	1-OH-24	$C_{24}H_{50}O$	2612
**Aldehyde**			
2,2 - Dimethylpentanal	-	$C_7H_{14}O$	1341
**Phenolics**			
4-Hydroxy-3-methoxy-benzaldehyde (Vanillin)	-	$C_8H_8O_3$	1518
4-Hydroxy-3,5-dimethoxy-benzaldehyde (Syringaldehyde)	-	$C_9H_{10}O_4$	1709
4-(2-Hydroxyethyl)-2-methoxyphenol (Homovanillyl alcohol)	-	$C_9H_{12}O_3$	1769
**Hydroxycinnamic acids**			
3-(2-Hydroxyphenyl)-2-propenoic acid (o-Coumaric acid)	-	$C_9H_8O_3$	1695
3-(4-Hydroxyphenyl)-2-propenoic acid (p-Coumaric acid)	-	$C_9H_8O_3$	1789
2-Propenoic acid, 3-(4-hydroxy-3-methoxyphenyl)-, methyl ester (Methyl ferulate)			1973
3-(2,3,4-Trimethoxyphenyl) propenoic acid (2,3,4-Trimethoxycinnamic acid)	-	$C_{12}H_{14}O_5$	2026
3-(3,4,5-Trimethoxyphenyl)-2 -propenoic acid (Methyl sinapate)	-	$C_{12}H_{14}O_5$	2158
3-(4-Hydroxy-3,5-dimethoxyphenyl)-2-propenoic acid (Sinapic acid)	-	$C_{11}H_{12}O_5$	2260
**Fatty acids**			
4-Oxopentanoic acid (Levulinic acid)	4-oxo-5:0	$C_5H_8O_3$	1495
Nonanedioic acid (Azelaic acid)	9:0-diacid	$C_9H_{16}O_4$	1543
Tetradecanoic acid (Myristic acid)	14:0	$C_{14}H_{28}O_2$	1721
Unknown fatty acid 1	79(100), 91(66), 93(52), 67(38), 77(35), 41(33), 105(33)		1889
(7*E*,10*E*)-Hexadeca-7,10-dienoic acid	7t10t-16:2	$C_{16}H_{28}O_2$	1895
(7Z,10Z,13Z)-Hexadeca-7,10,13 -trienoic acid (Roughanic acid)	7c10c13c-16:3	$C_{16}H_{26}O_2$	1905
(Z)-9-Hexadecenoic acid (Palmitoleic acid)	9c-16:1	$C_{16}H_{30}O_2$	1910
Hexadecanoic acid (Palmitic acid)	16:0	$C_{16}H_{32}O_2$	1931
Unknown fatty acid 2	75(100), 117(84), 285(32), 55(19), 41(19), 71(17), 211(14)		2047
(9Z,12Z,15Z)-Octadeca-9,12,15-trienoic acid (α-Linolenic acid)	9c12c15c-18:3	$C_{18}H_{30}O_2$	2091
(9Z,12Z)-Octadeca-9,12-dienoic acid (Linoleic acid)	9c12c-18:2	$C_{18}H_{32}O_2$	2102
(Z)-9-Octadecenoic acid (Oleic acid)	9c-18:1	$C_{18}H_{34}O_2$	2113
(E)-9-Octadecenoic acid (Elaidic acid)	9t-18:1	$C_{18}H_{34}O_2$	2124
Octadecanoic acid (Stearic acid)	18:0	$C_{18}H_{36}O_2$	2135
Hexadecanedioic acid (Thapsic acid)	16:0-diacid	$C_{16}H_{30}O_4$	2260
Unknown fatty acid 3	43(100), 98(87), 55(86), 69(56), 83(50), 41(43), 79(36)	-	2272
Icosanoic acid (Arachidic acid)	20:0	$C_{20}H_{40}O_2$	2342
Unknown fatty acid 4	98(100), 43(90), 55(76), 74(59), 69(54), 83(50),	-	2348

	236(17), 255(17)		
Octadecadienedioic acid	18:2-diacid	$C_{18}H_{30}O_4$	2425
Unknown fatty acid 5	55(100), 81(78), 67(66), 41(54), 95(52), 276(47), 309(23)		2438
Octadecanedioic acid	18:0-diacid	$C_{18}H_{32}O_4$	2462
Unknown fatty acid 6	91(100), 79(95), 43(85), 106(78), 67(64), 119(51), 133(35), 290(13)		2487
Unknown fatty acid 7	67(100), 79(91), 43(82), 93(71), 121(58), 55(57), 320(14), 292(12)		2499
9,10-Dihydroxyoctadecanoic acid (9,10-Dihydroxystearic acid)	9,10-diOH-18:0	$C_{18}H_{36}O_4$	2511
(11*E*,14*E*)-Icosa-11,14-dienoic acid	11t14t-20:2	$C_{20}H_{36}O_2$	2516
Docosanoic acid (Behenic acid)	22:0	C22H44O2	2536
(Z)-15-Tetracosenoic acid (Nervonic acid)	15c-24:1	$C_{24}H_{46}O_2$	2714
Tetracosanoic acid (Lignoceric acid)	24:0	$C_{24}H_{48}O_2$	2727
Pentacosanoic acid (Hyenic acid)	25:0	$C_{25}H_{50}O_2$	2820
Hexacosanoic acid (Cerotic acid)	26:0	$C_{26}H_{52}O_2$	2927
(Z)-13-Docosenoic acid (Erucic acid)	13c-22:1	C22H42O2	2927
Octacosanoic acid (Montanic acid)	28:0	$C_{28}H_{56}O_2$	3150
**Fatty acid ester**			
(10Z, 12Z)-9-Methyl- 10,12-hexadecadien-1 -yl acetate (9-Methyl-Z,Z- 10,12-hexadecadien- 1-ol acetate)	9Me- 10c12c-16:2-acet	$C_{19}H_{34}O_2$	2586
**Monoglycerides**			
Hexadecanoic acid, 2,3-dihydroxypropyl ester (1-Monopalmitin)	MG(16:0/0:0/0:0)	$C_{19}H_{38}O_4$	2676
Octadecanoic acid, 2-hydroxy-1-(hydroxymethyl) ethyl ester (2-Monostearin)	MG(0:0/18:0/0:0)	$C_{21}H_{42}O_4$	2860
**Indoles**			
1H-Indol-3-ylacetonitrile (3 -Indoleacetonitrile)		$C_{11}H_9N$	1819
1H-Indol-3-ylacetic acid (Indole-3-acetic acid)		$C_{10}H_9NO_2$	1829
1H-Indole-3-carboxylic acid (3-Indolecarboxylic acid)		$C_9H_7NO_2$	1941
2-(5-Methoxy-1H-indol-3-yl) acetic acid (5-Methoxyindol-3-ylacetic acid)		$C_{11}H_{11}NO_3$	1973
(4-Methoxy-1H-indol-3-yl) acetonitrile (Arvelexin)		$C_{11}H_{10}N_2O$	2026
Unknown indole 1	160(100), 219(53), 130(39), 117(9), 89(9), 103(6)	-	2037
Unknown indole 2	130(100), 204(88), 118(51), 189(42), 145(21), 171(16)	-	2091
2-(5-Methoxy-1H-indol-3-yl) ethanol (5 -Methoxytryptophol)	-	$C_{11}H_{13}NO_2$	2180
Unknown indole 3	160(100), 234(85), 175(58), 130(29), 219(21), 117(14)	-	2283
**Polyol**			
1,2,3-Propanetriol (Glycerol)		$C_3H_8O_3$	1334
**Carbohydrate**			
2,6 -Anhydro-D-fructofurano se (2,6-Anhydrofructose)		$C_6H_{10}O_5$	1849
**Diterpene alcohol**			
(2*E*,7*R*,11*R*)-3,7,11,15-Tetramethyl-2-hexadecen-1-ol (Phytol)		$C_{20}H_{40}O$	2126

Table S3. Compounds detected by LC-QTOF-MS (ESI negative mode) in leaves of wild- type, *tggl, tgg2* single and *tggl tgg2* double mutants of *Arabidopsis* through untargeted metabolic profiling.

Compounds	Measured mass (m/z)	Calculated mass (m/z)	Difference measured vs. calculated (ppm)	Molecular structure	Retention time (RT) min
**Glucosinolates (GSLs)**					
Glucoiberin (3-Methylsulfinylpropyl GSL)	422.0253	422.0254	-0.51	$C_{11}H_{21}NO_{10}S_3$	3.40
Glucoraphanin (4-Methylsulfinylbutyl GSL)	436.0365	436.0411	-10.56	$C_{12}H_{23}NO_{10}S_3$	3.70
Glucoalyssin (5-Methylsulfinylpentyl GSL)	450.0569	450.0568	0.22	$C_{13}H_{25}NO_{10}S_3$	5.23
Glucoibarin (7-Methylsulfinylheptyl GSL)	478.0879	478.0880	-0.22	$C_{15}H_{29}NO_{10}S_3$	14.12
Glucoerucin (4-Methylthiobutyl GSL)	420.0463	420.0462	0.18	$C_{12}H_{23}NO_9S_3$	16.85
Glucohirsutin (8-Methylsulfinyloctyl GSL)	492.1037	492.1037	-0.14	$C_{16}H_{31}NO_{10}S_3$	19.80
Hexyl glucosinolatet	402.0895	402.0898	-0.80	$C_{13}H_{25}NO_9S_2$	29.62
Glucobrassicin (Indol-3-ylmethyl GSL)	447.0533	447.0537	-0.90	$C_{16}H_{20}N_2O_9S_2$	19.53
Neoglucobrassicin (1-Methoxy-indol-3-yl-methyl GSL)	477.0625	477.0643	-3.73	$C_{17}H_{22}N_2O_{10}S_2$	25.11
**Flavonol glycosides**					
Kaempferol 3-O-rhaglu 7-O-rha (Kaempferol 3-O-rhamnosyl- glucoside 7-O-rhamnoside)	739.2089	739.2091	-0.24	$C_{39}H_{50}O_{24}$	19.29
Kaempferol-3 -glu-7-rha (Kaempferol-3 -O-glucoside-7-O-rhamnoside)	593.1503	593.1511	-1.46	$C_{27}H_{30}O_{15}$	23.54
Kaempferol-soph-glu-coumaroyl (Kaempferol-3 -O-(p-coumaroyl)sophoroside 7-O- glucoside)	917.2347	917.2357	-1.06	$C_{42}H_{46}O_{23}$	26.53
Quercetin 7-O-rha-3-rhaglu (Quercetin 7-O-rhamnoside 3-O-rhamnosylglucoside)	755.2053	755.2040	1.73	$C_{33}H_{40}O_{21}$	17.74
Quercetin 3-O-rha 7-O-glu (Quercetin 3-O-rhamnoside 7-O-glucoside)	609.1461	609.1461	-0.08	$C_{27}H_{30}O_{16}$	21.12
**Sinapoyl esters**					
Sinapoylglucose	385.1146	385.1140	1.57	$C_{17}H_{22}O_{10}$	16.95
Sinapoylmalate	223.0603	223.0603	0.22	$C_{15}H_{16}O_9$	26.45
Sinapoyl-caffeoylquinic acid methyl ester	591.1713	591.1719	-1.12	$C_{27}H_{28}O_{13}$	31.41
Sinapoyl-caffeoylquinic acid methyl ester (isomer)	591.1708	591.1719	-1.74	$C^{27}H_{28}O_{13}$	34.56

t Hexyl glucosinolate identified on basis of elemental composition and from mass as reported before (Beekwilder et al. 2008 and Matsuda et al. 2010).
